# Dietary polyunsaturated fatty acids and incidence of end-stage renal disease in the Southern Community Cohort Study

**DOI:** 10.1186/s12882-016-0371-y

**Published:** 2016-10-18

**Authors:** Rakesh Malhotra, Kerri L. Cavanaugh, William J. Blot, T. Alp Ikizler, Loren Lipworth, Edmond K. Kabagambe

**Affiliations:** 1 Division of Nephrology and Hypertension, Department of Medicine, Vanderbilt University Medical Center, Nashville, TN 37232 USA; 2 Vanderbilt Center for Kidney Disease, Nashville, TN 37232 USA; 3 Division of Epidemiology, Department of Medicine, Vanderbilt University Medical Center, 2525 West End Ave, Suite 800, Nashville, TN 37203 USA; 4 International Epidemiology Institute, Rockville, MD 20850 USA; 5 Present address: Division of Nephrology and Hypertension, University of California San Diego, San Diego, CA 92161 USA

**Keywords:** End-stage renal disease, Polyunsaturated fatty acids

## Abstract

**Background:**

Whether polyunsaturated fatty acids (PUFA) are associated with end-stage renal disease (ESRD) in populations with a high burden of risk factors for kidney disease is unknown. We sought to determine whether PUFA intake is associated with ESRD.

**Methods:**

We conducted a nested case–control study of ESRD within the Southern Community Cohort Study (SCCS), a prospective cohort of low-income blacks and whites in the southeastern US (2002–2009). Through 2012, 1,074 incident ESRD cases were identified by linkage with the United States Renal Data System and matched to 3,230 controls by age, sex and race. Dietary intake of total, n-3 or n-6 PUFA was assessed from a validated food frequency questionnaire administered at baseline. Odds ratios (ORs) and 95 % confidence intervals (CIs) were computed from logistic regression models that included matching variables, body mass index, smoking, diabetes, hypertension, education, income, total energy intake and percent energy from protein and saturated fat.

**Results:**

The mean (SD) age of participants was 55 (9) years. Most participants were women (55 %), black (87 %), with hypertension (67 %) and on average obtained 8 % of their energy from PUFA. Higher PUFA intake was marginally associated with a lower risk of ESRD in adjusted analyses. The adjusted odds ratios (95 % confidence intervals) for ESRD for the 5^th^ vs. 1^st^ quintile of PUFA were 0.79 (0.60–1.05; *P*_trend_ = 0.06) for total PUFA, 0.81 (0.61–1.06; *P*_trend_ = 0.04) for n-6 PUFA and 0.93 (0.71–1.21; *P*_trend_ = 0.45) for n-3 PUFA.

**Conclusions:**

We observed a marginally significant inverse trend between dietary PUFA intake and ESRD incidence, mainly driven by n-6 fatty acid intake. Our findings require replication but suggest that a diet rich in n-6 PUFA may prevent ESRD development in a population with a high burden of kidney disease risk factors.

## Background

End-stage renal disease (ESRD) is major cause of morbidity and mortality [1–4] in the United States and other countries. While several studies have established diabetes and hypertension as strong risk factors for development of chronic kidney disease (CKD) and progression to ESRD [5–8], modifiable lifestyle factors such as diet may also be important contributors. Over the past decade, results from experimental and clinical studies have provided evidence that higher intake of polyunsaturated fatty acids (PUFA) is associated with lower risk of hypertension [9], diabetes progression [10] and in some [7, 11–15] but not all [15–17] studies, with a lower risk of developing CKD and its progression to ESRD. Most studies enrolled primarily affluent populations and had relatively small sample sizes, limiting their generalizability to lower socioeconomic populations who tend to have lower PUFA intake and a heavy burden of risk factors for kidney disease.

While most studies on PUFA and kidney diseases have focused on n-3 fatty acids [15], numerous large human studies have shown that dietary or tissue levels of n-6 PUFA are inversely associated with inflammation [18–24] and with clinical events such as blood pressure, diabetes, and cardiovascular diseases (CVD) [25, 26], conditions that predispose to or complicate ESRD. In the Nurses’ Health Study (*n* = 83,349 women) [27], the hazard ratio (HR) (95 % confidence interval) for all-cause mortality was 0.85 (0.81–0.89) when comparing the top to the lowest quintile of n-6 PUFA. The corresponding HR for CVD mortality was 0.89 (0.85–0.94). An inverse association with mortality was also observed for arachidonic acid, the n-6 PUFA previously thought to lead to more pro-inflammatory products than anti-inflammatory products (HR = 0.90; 95 % CI: 0.85–0.94). Studies that aim to understand the distinct contributions of n-3 and n-6 fatty acids are important because these fatty acid series and their subtypes vary in their biological potencies. For instance, in our previous studies with erythrocyte fatty acids, n-6 fatty acid levels showed stronger inverse associations with lipids, blood pressure and markers of insulin resistance than n-3 fatty acids [28]. Others have also shown differences between n-6 and n-3 fatty acids in relation to markers of inflammation, with consistent reduction in inflammation observed for n-6 fatty acids [29]. Similarly, in hemodialysis patients, benefits on inflammation and mortality are observed for n-6 but not n-3 fatty acids [15]. Despite inconsistencies in the associations between n-3 and n-6 fatty acids with inflammation and kidney disease, some studies have reported benefits on kidney function from both n-3 and n-6 fatty acids [14].

Current dietary guidelines for the general population recommend substituting trans- and saturated fatty acids with PUFA [30] partly because of the beneficial effects of PUFA on blood pressure and blood lipid profiles [31, 32]. However, with only limited large population data on the relation between dietary fat and kidney diseases, Kidney Disease Outcomes Quality Initiative (KDOQI) guidelines for nutrition in CKD have not included specific dietary fatty acid intake recommendations for the prevention or management of kidney diseases [33].

In the present study, nested in a large prospective cohort of 86,000 low-income blacks and whites with a high burden of kidney disease risk factors, we sought to determine whether total intake of PUFA is associated with incidence of ESRD and whether the association varied by type of PUFA consumed (i.e., n-3 vs. n-6-PUFA). We have previously shown that the age-adjusted incidence rate of ESRD in this cohort, with 329,003 person-years of follow-up through 2012, was 214 per 100,000 person-years [34]. We hypothesized that higher intake of PUFA will be associated with a lower risk of ESRD and that both n-3 and n-6 fatty acids would be associated with a lower risk of ESRD.

## Methods

### The Southern Community Cohort Study

The sample for this nested case–control study was derived from individuals enrolled in the Southern Community Cohort Study (SCCS). The SCCS is a large ongoing population-based prospective cohort study examining health disparities among 86,000 black and white men and women, age 40–79, from 12 southeastern states (Alabama, Arkansas, Florida, Georgia, Kentucky, Louisiana, Mississippi, North Carolina, South Carolina, Tennessee, Virginia, and West Virginia) enrolled between 2002 and 2009. Approximately 86 % of participants were recruited in-person from one of the participating community health centers (CHC), which provide primary health care for low-income and under-insured populations [35, 36], while the remaining 14 % were recruited from the general population by mail. The current analysis was restricted to CHC-enrollees, which ensured that participants were of similar socioeconomic status and had generally equal access to health care at cohort entry regardless of race. Detailed description of SCCS methods has been previously published and is available on the study website (http://www.southerncommunitystudy.org) [38]. All participants provided written informed consent, and the study was approved by Institutional Review Boards of Vanderbilt University Medical Center and Meharry Medical College. We adhered to the guidelines and methodology set forth in the STrengthening the Reporting of OBservational studies in Epidemiology Statement [38].

### Study population

All incident cases of ESRD among SCCS participants were identified by linking the cohort with the US Renal Data System (USRDS), which registers ESRD cases certified by a physician diagnosis and filed using a medical evidence report form (to the Medicare ESRD program) or when there is other evidence of chronic dialysis or a kidney transplant irrespective of the glomerular filtration rate (GFR) [39]. The data linkage spanned from January 1, 2002 to September 1, 2012, the latest date for which data were available, and since USRDS is a nationwide registry, there is near complete ascertainment of ESRD cases. SCCS participants who had a diagnosis of ESRD recorded in the USRDS prior to SCCS enrollment were excluded from our analyses [34]. Three controls were individually matched to each incident ESRD case by age at cohort enrollment (±5-year categories), sex and race.

### Risk factor assessment

At enrollment in SCCS, CHC participant characteristics including demographic variables, socioeconomic status, medical history, and lifestyle attributes (e.g., smoking and physical activity) were assessed using a standardized computer-assisted personal interview (questionnaire available at http://www.southerncommunitystudy.org). Dietary intake was assessed at baseline using a validated food frequency questionnaire [35]. Estimates for nutrient intakes were calculated by utilizing sex- and race-specific nutrient databases derived from government food consumption surveys in the southern US [35, 40]. The overall dietary questionnaire has been extensively validated in the SCCS and other populations; while not specifically validated for PUFA, intakes of PUFA in the SCCS participants are similar to those of the general US population, as shown in our previous publications [37, 40].

## Statistical analyses

From the 4,788 participants in the age-, sex- and race-matched ESRD nested case–control study we excluded 484 individuals with missing data on covariates, leaving 1,074 incident ESRD cases and 3,230 controls for the final analyses. Since some of the case–control matches were broken because of missing data, to maximize use of data, we used methods for unmatched data while accounting for matching variables as covariates.

To reduce confounding by total energy intake, we expressed protein, saturated fat, monounsaturated fat and polyunsaturated fat intakes as percentages of daily energy intake and adjusted for daily total energy intake in the models. Next we tested whether the distributions of participant characteristics differed significantly by case–control status using the chi-square test for categorical variables and unpaired *t*-test for continuous variables. We further computed descriptive statistics for potential confounders by categories of major ESRD risk factors, namely hypertension and diabetes.

We then distributed participants into quintiles of energy intake from total, n-3 and n-6 PUFA and used unconditional logistic regression to estimate odds ratios (OR) and 95 % confidence intervals (CI) for the relation between intake of PUFAs and ESRD. For models with total, n-3 or n-6 PUFA intake as the independent variable, we adjusted for matching variables (age, sex and race), important predictors of ESRD in our cohort such as diabetes (yes or no) and hypertension (yes or no), as well as body mass index (BMI, kg/m^2^, continuous), education (< high school vs. high school or higher), income (< $ 15,000 vs. higher), smoking status (current and past vs. never), total energy intake and % energy from protein and saturated fat as continuous variables. For total, n-3 and n-6 PUFA variables, we computed median percent energy intake for each quintile and assigned it to all participants in a given quintile of the respective PUFA variable. The resulting continuous variable was added to the logistic regression model to obtain the *p*-value for trend.

In a subset of the study sample for whom baseline serum creatinine measurements were available, we conducted sensitivity analyses to examine whether baseline kidney function confounded the association between PUFA intake and ESRD. We performed logistic regression analyses in a subgroup of 1,574 participants with eGFR ≥ 60 mL/min/1.73 m^2^ at baseline. These analyses included 180 incident ESRD cases. All analyses were conducted using SAS version 9.4 (SAS Institute, Cary, NC). *P*-values ≤0.05 were considered statistically significant.

## Results

The characteristics of ESRD cases and controls are shown in Table 1. The age, sex and race distributions of cases and controls were similar given the matched design (54.6 ± 9.0 vs 54.6 ± 8.8 years, 54.8 vs 55.4 % women, and 86.3 vs 86.9 % black, respectively). Participants in the SCCS who subsequently developed ESRD were more likely to have history of diabetes (63.9 vs 23.0 %; *P* <0.0001) and hypertension (84.2 vs 61.3 %; *P* <0.0001). Compared to controls, ESRD cases were also likely to have higher BMI (31.8 ± 8.2 vs 30.3 ± 7.2; *P* <0.0001) and a household income of < $15,000 (68.5 vs 59.7 %; *P* <0.0001).

**Table 1:** Characteristics of end-stage renal disease cases and controls from the Southern Community Cohort Study

Variable	ESRD cases (*n* = 1,074)	Controls (*n* = 3,230)	*P*
Age at enrollment, years^a^	54.6 ± 9.0	54.6 ± 8.8	--
Sex, % women^a^	54.8	55.4	--
Race, % black^a^	86.3	86.9	--
Body mass index, kg/m^2^	31.8 ± 8.2	30.3 ± 7.2	<0.0001
Smoking status, %			0.13
Current smoker	37.6	40.9	
Past smoker	26.6	24.3	
Never smoker	35.8	34.7	
Education < high school, %	37.0	34.4	0.13
Household income < $15,000/year, %	68.5	59.7	<0.0001
History of hypertension, % yes	84.2	61.3	<0.0001
History of diabetes, % yes	63.9	23.0	<0.0001

*Abbreviations: ESRD* end-stage renal disease

Continuous variables are expressed as mean ± SD and categorical variables as percentage (%)

^a^Matching variable

Table 2 shows baseline dietary characteristics of study participants stratified by diabetes, hypertension and ESRD status. Individuals with diabetes reported consuming significantly more protein and PUFA as a percent of total energy than those without diabetes (*P* < 0.0001). Among participants with diabetes but without hypertension, both total and n-6 PUFA intake were lower among ESRD cases than controls but the differences in other groups were not pronounced. Regardless of hypertension status, ESRD cases with diabetes had lower dietary fiber intakes than individuals with diabetes but without ESRD. The stratified groups were similar with regard to dietary carbohydrate, saturated fatty acids (SFAs), mono-unsaturated fatty acid (MUFAs) and fiber intake (Table 2).

**Table 2:** Characteristics of end-stage renal disease cases and controls from the Southern Community Cohort Study by hypertension and diabetes

	No hypertension				Hypertension			
	No diabetes		Diabetes		No diabetes		Diabetes	
	ESRD	No ESRD	ESRD	No ESRD	ESRD	No ESRD	ESRD	No ESRD
N	91	1112	79	139	297	1374	607	605
Age at enrollment, y	49.8 ± 8.4	51.6 ± 8.4	54.6 ± 9.3	54.3 ± 7.9	54.6 ± 9.7	55.5 ± 8.4	55.2 ± 8.5	58.2 ± 8.6
Sex, % women	36.3	46.5	65.8	54.7	43.4	57.6	61.8	66.6
Race, % black	82.4	85.2	86.1	87.1	89.2	87.6	85.5	88.3
Smoking, % current	72.5	51.4	31.7	38.1	48.8	39.9	27.7	24.6
Body mass index, kg/m^2^	27.0 ± 6.0	27.6 ± 6.2	30.8 ± 7.0	31.7 ± 7.0	30.2 ± 8.0	30.8 ± 7.2	33.5 ± 8.3	33.5 ± 7.0
Total energy, kcal/d	3358 ± 1737	2810 ± 1559	2313 ± 1344	2429 ± 1379	2693 ± 1473	2516 ± 1384	2203 ± 1299	2241 ± 1325
Carbohydrate, % energy	49.0 ± 8.6	48.7 ± 8.8	50.0 ± 9.5	48.9 ± 9.0	49.8 ± 10.0	50.2 ± 9.2	49.7 ± 8.5	50.0 ± 9.0
Protein, % energy	14.8 ± 3.0	14.7 ± 3.0	16.1 ± 3.1	15.9 ± 3.6	14.8 ± 3.2	14.9 ± 3.0	16.2 ± 3.3	15.8 ± 3.1
MUFA, % energy	13.0 ± 2.4	13.0 ± 2.6	12.8 ± 2.7	13.4 ± 2.9	12.8 ± 2.7	12.7 ± 2.7	13.1 ± 2.5	13.0 ± 2.7
SFA, % energy	10.0 ± 1.9	10.1 ± 2.2	10.1 ± 2.3	10.3 ± 2.3	10.0 ± 2.3	9.9 ± 2.3	10.2 ± 2.1	10.0 ± 2.3
PUFA, % energy	7.8 ± 1.7	7.9 ± 1.7	7.8 ± 1.8	8.5 ± 2.0	7.8 ± 1.7	7.9 ± 1.8	8.2 ± 1.7	8.2 ± 1.8
N3-PUFA, % energy	0.07 ± 0.09	0.06 ± 0.07	0.08 ± 0.07	0.07 ± 0.07	0.07 ± 0.09	0.07 ± 0.07	0.07 ± 0.08	0.07 ± 0.07
N6-PUFA, % energy	7.5 ± 1.6	7.6 ± 1.7	7.6 ± 1.7	8.2 ± 2.0	7.5 ± 1.6	7.6 ± 1.7	7.9 ± 1.6	7.9 ± 1.8
Dietary fiber, g/d	25.7 ± 14.5	22.5 ± 13.5	20.6 ± 11.9	22.7 ± 13.0	21.9 ± 13.0	21.3 ± 12.9	20.6 ± 12.2	21.4 ± 12.7

*Abbreviations:* *ESRD* End-stage renal disease, *MUFA* Monounsaturated fatty acids, *SFA* Saturated fatty acids, *PUFA* Polyunsaturated fatty acids

Continuous variables are expressed as mean ± SD and categorical variables as percentage (%)

As shown in Table 3, median intakes for n-3 PUFA were low while median total and n-6 PUFA intakes were similar to those of the general US population (about 8 % of energy). In the final adjusted multivariable logistic regression models, we observed marginally significant modest inverse associations between total PUFA and n-6 PUFA and ESRD incidence. For instance, the adjusted ORs (95 % CIs) for ESRD for the 5^th^ vs. 1^st^ quintile of PUFA were 0.79 (0.60–1.05; *P*_trend_ = 0.06) for total PUFA and 0.81 (0.61–1.06; *P*_trend_ = 0.04) for n-6 PUFA. Although the OR estimates for n-3 PUFA were in the same direction as those for total and n-6 PUFA, there was no evidence for an inverse trend between n-3 PUFA and ESRD incidence (OR = 0.93; 95 % CI: 0.71–1.21; *P*_trend_ = 0.45) (Table 3).

**Table 3:** Odds ratios and 95 % confidence intervals for the associations between total, n-6 and n-3 polyunsaturated fatty acids and incident end-stage renal disease in a case–control study nested within the Southern Community Cohort Study

Fatty acid	Quintiles^a^					*P* for trend
	1 (Lowest)	2	3	4	5 (Highest)	
Total PUFA						
ESRD cases	208	207	233	212	214	
Median, % energy	5.8	7.1	8.0	8.8	10.2	
Model 1	1.00	1.00 (0.80–1.25)	1.18 (0.95–1.47)	1.04 (0.83–1.30)	1.04 (0.84–1.31)	0.63
Model 2	1.00	0.94 (0.73–1.20)	0.99 (0.77–1.27)	0.80 (0.62–1.04)	0.76 (0.59–0.99)	0.02
Model 3^b^	1.00	0.94 (0.73–1.21)	1.01 (0.78–1.32)	0.85 (0.65–1.12)	0.79 (0.60–1.05)	0.06
n-6 PUFA						
ESRD cases	205	217	235	206	211	
Median, % energy	5.62	6.89	7.70	8.52	9.79	
Model 1	1.00	1.08 (0.87–1.35)	1.22 (0.98–1.52)	1.02 (0.82–1.28)	1.04 (0.84–1.31)	0.88
Model 2	1.00	1.02 (0.80–1.31)	1.02 (0.79–1.31)	0.80 (0.62–1.04)	0.78 (0.60–1.01)	0.01
Model 3^b^	1.00	1.02 (0.80–1.32)	1.03 (0.80–1.34)	0.85 (0.64–1.11)	0.81 (0.61–1.06)	0.04
n-3 PUFA						
ESRD cases	223	204	207	208	232	
Median, % energy	0.01	0.03	0.05	0.08	0.15	
Model 1	1.00	0.89 (0.72–1.11)	0.91 (0.73–1.13)	0.91 (0.73–1.14)	1.06 (0.85–1.32)	0.66
Model 2	1.00	0.90 (0.71–1.14)	0.85 (0.67–1.08)	0.86 (0.67–1.10)	0.92 (0.71–1.19)	0.38
Model 3^b^	1.00	0.89 (0.70–1.14)	0.85 (0.67–1.09)	0.86 (0.67–1.11)	0.93 (0.71–1.21)	0.45

*Abbreviations: ESRD* End-stage renal disease, *PUFA* Polyunsaturated fatty acids

^a^Values are odds ratios and corresponding 95 % confidence intervals from models in which total, n-6 and n-3 PUFA were modeled separately. Model 1 included matching variables (age, sex and race), total energy intake and quintiles of energy from total PUFA or n-3 PUFA or n-6 PUFA. Model 2 additionally adjusted for diabetes and percent energy from protein intake. Model 3 additionally adjusted for body mass index, hypertension, education level, household income, smoking status and percent energy from saturated fat intake

^b^In a sensitivity analysis among 1,574 participants with eGFR ≥60 mL/min/day (180 cases and 1394 controls), further adjusting for eGFR did not change the results appreciably. For example, in analyses adjusting for eGFR and covariates in model 3 and using the lowest quintile as the referent, the ORs (95 % CIs) for ESRD in the 2^nd^, 3^rd^, 4^th^ and 5^th^ quintile of total PUFA were 0.90 (0.485, 1.69), 1.32 (0.71, 2.46), 1.44 (0.75, 2.75) and 0.90 (0.47, 1.74), respectively. The corresponding OR (95 % CIs) for n-6 PUFA were 0.79 (0.42, 1.47), 1.26 (0.68, 2.30), 1.11 (0.58, 2.14) and 0.82 (0.43, 1.57) while they were 0.93 (0.53, 1.61), 0.55 (0.30, 1.02), 0.72 (0.40, 1.31) and 1.02 (0.56, 1.86) for n-3 PUFA

## Discussion

In the current prospective study among black and white men and women of generally low socioeconomic status and with a high burden of risk factors for kidney disease, we have demonstrated that higher intake of PUFA, specifically n-6 PUFA, is associated with a modestly lower incidence of ESRD independent of established risk factors such as diabetes and hypertension.

Prior observational studies have reported a protective effect of dietary PUFA intake on renal dysfunction [13, 14]. In the Blue Mountains Eye Study, Gopinath et al. showed that a diet rich in n-3 PUFA was significantly associated with reduced likelihood of having CKD (i.e., having a GFR <60 mL/min per 1.73 m^2^; adjusted OR = 0.87; 95 % CI: 0.76–0.99) among 2,600 participants aged > 49 years [13]. Similarly, in the InCHIANTI (Aging in the Chianti Area) study of over 931 participants aged ≥ 65 years, researchers found an inverse association between total plasma PUFA levels and age-associated decline in renal function, during 3-year follow up [14]. In addition, they showed an inverse relation between total plasma PUFA and urine protein excretion [14]. Meta-analyses examining the effect of fish-oil in CKD secondary to IgA nephropathy, diabetic nephropathy and/or systemic lupus erythematosus showed reduction in albumin excretion and a trend towards slowing rate of decline in GFR [16]. In the Alpha Omega trial, long-term supplementation with the n-3 fatty acids eicosapentaenoic acid and docosahexaenoic acid provided beneficial effects on kidney function among patients with a history of myocardial infarction [41]. Among renal transplant patients, high levels of plasma marine n-3 PUFA have been associated with reduced risk of graft loss [42], and supplementation with an n-3 PUFA-rich diet showed reduction of systemic inflammation and proteinuria [43].

In our study, we found a statistically significant trend for an inverse association between dietary n-6 PUFA intake and incidence of ESRD while a non-significant association was detected for n-3 PUFA. This observation for ESRD is in line with findings from a previous study in dialysis patients [15] in which dietary n-6 PUFA intake was associated with reduction in all-cause mortality but a null association was observed between n-3 PUFA and inflammation or mortality [15]. In the GO-FISH study, a single-center randomized, placebo-controlled, two-period crossover trial involving 29 adult diabetic patients with micro- or macroalbuminuria, 4 g/d of n-3 PUFA for 6 weeks showed a non-significant decrease in urinary albumin excretion (−7.2 %; 95 % CI −20.6 to 8.5; *P* = 0.35) and a significant effect on urine neutrophil gelatinase-associated lipocalin (NGAL) excretion (−16 %; 95 % CI −29.1 to −0.5 %; *P* = 0.04) [44]. The investigators performed a subgroup analysis among patients on renin angiotensin aldosterone system (RAAS) inhibitors and found that n-3 PUFA supplementation had significant protective effects on multiple markers of kidney injury [44]. These data, though inconsistent, may suggest that n-6 PUFA and probably n-3 PUFA may contribute to both the development of ESRD and outcomes (e.g., CVD) of kidney diseases.

Dietary PUFA may be reno-protective through a variety of mechanisms. Literature from experimental studies suggests that high PUFA levels may preserve renal morphology by modifying kidney inflammatory and proliferative responses [18, 19, 45]. In vitro studies show that PUFA may decrease mesangial cell proliferation through reduced activity of platelet-derived growth factor [45]. The reno-protective effects of PUFA may also occur through reduction of inflammation since PUFA downregulate NF-κB, inhibit expression of leukocyte adhesion molecules and modify intrarenal and glomerular hemodynamics through decreased synthesis of renal prostaglandin like thromboxane A2 [18, 19]. Another plausible explanation for reno-protection is that adequate levels of PUFA may reduce factors (e.g., blood pressure, oxidative stress, endothelial dysfunction and vascular calcification) that are involved in the pathogenesis of kidney diseases [25, 26].

The US Institute of Medicine of the National Academies of Science recommends that the daily dietary allowance for n-3 PUFA and n-6 PUFA be 0.6–1.2 % and 5–10 % of total energy intake, respectively [46]. Compared with these recommended intakes, PUFA intake in our study exceeds 9 % of energy only in the highest quintile of intake of n-6 and total PUFA, which could in part explain why the risk of ESRD was not reduced in the lower quintiles. No guidelines exist for dietary intake of PUFA in CKD/ESRD patients, and our results suggest a need for further evaluation of PUFA intake or blood levels in relation to kidney disease prevention and management, which may inform policy on whether it is important to include PUFA in dietary guidelines.

Our study has several strengths. First, the SCCS is a unique large population-based cohort comprising black and white participants of comparable low socioeconomic status and with a high burden of risk factors for kidney disease, a population not adequately represented in previous studies. Moreover, detailed information on ESRD risk factors, diet and confounders was obtained at baseline, and incident ESRD was ascertained using the USRDS for the entire cohort in a complete and systematic manner, thereby minimizing the possibility for selection bias. In addition, the questionnaire used to record dietary intake has been extensively validated in the SCCS population and other populations in the US [35, 40], and the study used energy-adjusted macronutrient variables, thus providing a more accurate estimate of the association between PUFA intake and ESRD.

There are, however, some limitations to our study. First, our study cannot prove causality and it is difficult to rule out residual confounding despite adjustment for several important confounders. As shown in analyses stratified by hypertension and diabetes, the distribution of common lifestyle variables was similar between ESRD cases and controls, suggesting that the impact of confounding by these variables is likely minimal. The relatively lower dietary n-3 PUFA intakes in SCCS participants may have precluded detecting its beneficial effect on renal function as postulated in other studies [13, 15]. Secondly, FFQ data were self-reported once at baseline and no updated dietary intakes were obtained so as to study effects of long-term, sustained PUFA intake. The questionnaire was not validated specifically for PUFA intake, and there could be reporting errors in the assessment of PUFA; however, they are likely to be minimal since, as we have shown in previous studies, estimated intakes of PUFA in the current study are similar to those of the general US population (~8 % of energy) reported from NHANES and other studies [47]. Our FFQ collected dietary history for the previous 12 months and it is possible that a participant’s diet at baseline may have changed during follow-up. Lastly, levels of serum creatinine as a marker of kidney function at baseline and/or follow up are only available for less than half of the study participants, thus precluding our ability to fully account for baseline differences in kidney function in our analyses. Nonetheless, the inverse association between PUFA intake and ESRD was still apparent after adjusting for eGFR in a subgroup with eGFR ≥60 mL/min/1.73 m^2^.

## Conclusions

We observed a marginally significant inverse trend between PUFA intake and ESRD incidence that was mainly driven by n-6 fatty acid intake. Our findings suggest that a diet rich in n-6 PUFA may prevent ESRD development in a population with a high burden of kidney disease risk factors. Further studies that use plasma or red blood cell membrane PUFA levels to determine their role in ESRD incidence and whether they confer survival benefits are needed. It is important to establish the optimal individual-level dietary n-3 and n-6 PUFA intakes for use as prophylactic or therapeutic strategies for kidney disease.

## Abbreviations

BMI: Body mass index
CHC: Community health centers
CI: Confidence interval
CKD: Chronic kidney disease
CVD: Cardiovascular disease
eGFR: Estimated glomerular filtration rate
ESRD: End-stage renal disease
FFQ: Food-frequency questionnaire
KDOQI: Kidney Disease Outcomes Quality Initiative
MDRD: Modification of Diet in Renal Disease
OR: Odds ratio
PUFA: Polyunsaturated-fatty acids
SCCS: Southern Community Cohort Study
USRDS: United States Renal Data System
